# ﻿Karyotype of *Sabanejewiabulgarica* (Drensky, 1928) (Teleostei, Cobitidae) from the Danube Delta, Romania

**DOI:** 10.3897/compcytogen.17.103152

**Published:** 2023-07-05

**Authors:** Eva Hnátková, Zuzana Majtánová, Vendula Bohlen Šlechtová, Joerg Bohlen, Petr Ráb

**Affiliations:** 1 Department of Mathematics, Faculty of Engineering, Czech University of Life Sciences, 165 00 Prague, Kamýcká 129, Czech Republic Czech University of Life Sciences Prague Czech Republic; 2 Laboratory of Fish Genetics, Institute of Animal Physiology and Genetics, Academy of Sciences of Czech Republic, 277 21 Liběchov, Czech Republic Institute of Animal Physiology and Genetics, Academy of Sciences of Czech Republic Liběchov Czech Republic

**Keywords:** C-heterochromatin, Chromosome number, cobitoid loaches, karyotype structure

## Abstract

The karyotype of the freshwater fish *Sabanejewiabulgarica* (Drensky, 1928), from the Danube Delta, was studied by conventional Giemsa staining and the C-banding technique. The diploid chromosome number was 2n = 50. The karyotype contained 2 pairs of metacentric (the first one was much larger than the second one), 6 pairs of submetacentric and 17 pairs of subtelocentric to acrocentric chromosomes. Pericentromeric blocks of heterochromatin were revealed in most of the chromosome pairs. The karyotype phenotype of *S.bulgarica* was the same as found for *S.balcanica* from Northern Carpathian Mountains.

## ﻿Introduction

Freshwater fishes of the genus *Sabanejewia* Vladykov, 1929 are small (max 15 cm TL), have a benthic lifestyle in river habitats and can be distinguished from all other genera of Cobitidae by a specific sexual dimorphism (males with lateral body swellings) (Nalbant 1994; Kottelat and Freyhof 2007; Šlechtová et al. 2008). Their distribution spans from northern Italy to the Aral Sea basin including tributaries of the Black Sea, Caspian Sea, Baltic Sea, Aral Sea and Mediterranean Sea (Kottelat and Freyhof 2007). The highest taxonomic diversity was long time assumed to be in the Danube basin, but phylogenetic studies (Perdices and Doadrio 2001; Šlechtová et al. 2008; Vasil’eva et al. 2022) have shown that several phenotypes within this area are very closely related (*Sabanejewiabalcanica* (Karaman, 1922) species complex), so that the exact species composition is still not known.

*Sabanejewiabulgarica* (Drensky, 1928) was originally described as a species of the genus *Cobitis* Linnaeus, 1758 occurring in the lowest Danube basin including the Danube River itself. Junior synonyms are *C.albicoloris* Chichkoff, 1932 and *C.taeniatesselatus* Pietschman, 1937 (Kottelat 2012). For some time, most authors included *S.bulgarica* in the polytypic Balkan golden loach complex, with *Sabanejewiabalcanica*, as a subspecies (Nalbant 1957; Bănărescu 1964; Bănărescu et al. 1972). Vasil’eva and Vasil’ev (1988) analysed the geographical variations of characters among a number of local forms of golden loach throughout its range they showed that *bulgarica* is quite distinct from all other populations and deserves species rank (Nalbant 1994; Kottelat 1997, 2012). The main differences include the overall colour of body, character of Gambetta´s zone, pigmentation and character of spots on the end of the caudal peduncle and basis of caudal fin as well as the habitat, as it is the only deep-water riverine form of *Sabanejewia*. Recently, Križek et al. (2020) based on several lines of evidence and analysis of individuals from type localities of both *S.balcanica* and *S.bulgaricasensu*Bănărescu et al. (1972) have claimed that Danubian golden loaches are genetically closer to *S.bulgarica* and *S.balcanica* itself is to be restricted to the drainage of the Vardar River.

Chromosomes of the loaches of the genus *Sabanejewia* remain poorly studied. Ráb et al. (1991) summarized three previous studies dealing with *S.larvata* (De Filippi, 1859), *S.caspia* (Eichwald, 1838) and *S.kubanica* Vasil’eva et Vasil’ev, 1988. Vasil’ev and Vasil’eva (1994, 2019) reported karyotypes of *S.caspia* and *S.aurata* (De Filippi, 1863) from other locations (Kizylach Bay of the Caspian Sea, Kura River in Tbilisi and southern basin of the Bug River of the Black Sea, respectively). The karyotype of *S.baltica* Witkowski, 1994 from the Bug River in Poland was reported by Boroń (2000). All these studies document invariable 2n = 50 and remarkably small karyotype variability among the loaches of this genus. To contribute to the cytotaxonomy of *Sabanejewia*, the present report describes the karyotype and C-banding pattern of *S.bulgarica* from Danube Delta, Romania. Additionally, the comparison of karyotypes of all karyologically studied species of *Sabanejewia* was conducted.

## ﻿Materials and methods

**Figure 1.**
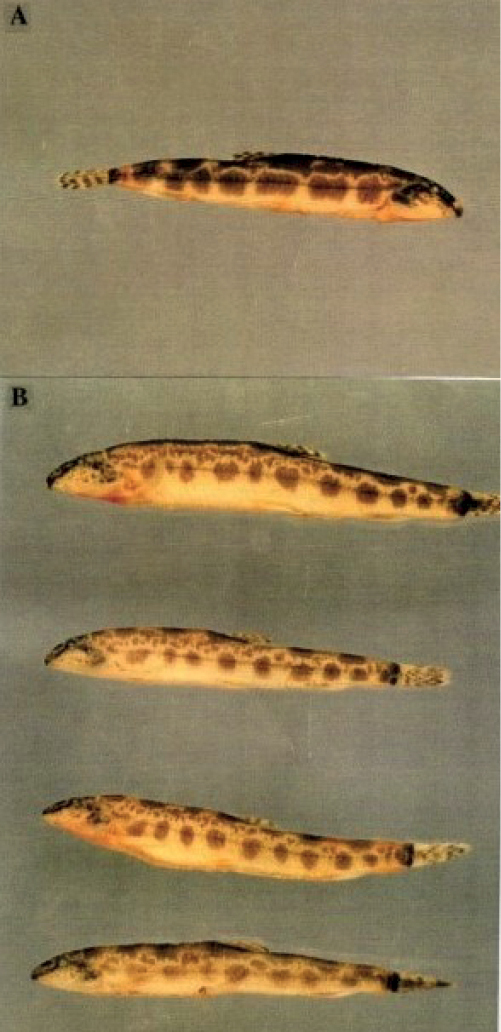
Analysed females of *Sabanejewiabulgarica* from the Saint George Branch, Danube Delta **A** individual with colour pattern and high body as described in original description by Drensky (1928)**B** individuals of *S.bulgarica* with other colour patterns.

The five *Sabanejewiabulgarica* specimens examined (all females) were collected in the Saint George Branch, Danube Delta (July 1997). The loach individuals arrived at the laboratory (Danube Delta Institute, Tulcea) in very bad condition and died subsequently. One female displayed the colour pattern and high body as described in original description by Drensky (1928), while four other individuals displayed other colour patterns (Fig. 1).

Standard procedure for chromosome preparation followed Ráb and Roth (1988) and C-banding technique followed Haaf and Schmid (1984). Chromosomes were classified according to the system of Levan et al. (1964). The analysed individuals are deposited as vouchers in fish collection of Laboratory of Fish Genetics, IAPG, AS CR, Liběchov (Accs. code. SB 7/97).

## ﻿Results and discussion

**Figure 2.**
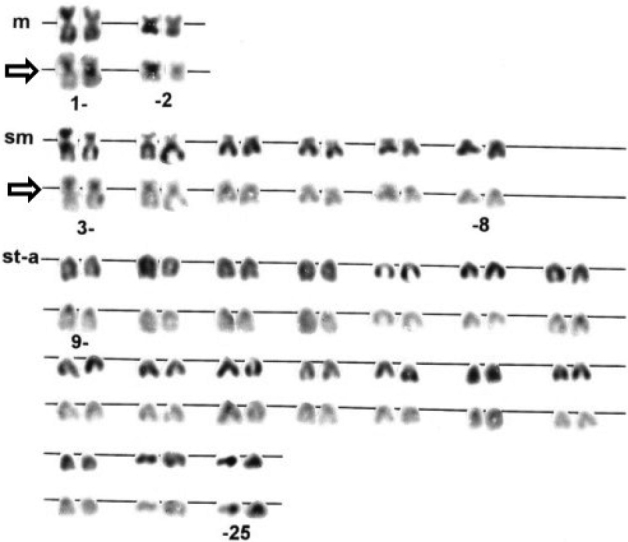
Karyotype of female *Sabanejewiabulgarica* arranged from conventionally Giemsa-stained (the first row) and sequentially C-banded (the second row, large blocks denoted by arrows) chromosomes. m – metacentric, sm – submetacentric, st – subtelocentric and a – acrocentric chromosomes.

The diploid chromosome number was determined as 2n = 50 in all five individuals. The detailed karyotype analysis was carried out on a single specimen with morphological characters corresponding to the original description (Fig. 1, top individual). The karyotype comprised 2 pairs of metacentric (m), 6 pairs of submetacentric (sm) and 17 pairs of subtelocentric (st) to acrocentric (a) chromosomes (Fig. 2). C-banding procedure reveals conspicuous pericentromeric blocks of heterochromatin in most of the chromosome pairs with very prominent blocks in the largest m chromosome and the first sm element.

Our results enable us to compare the karyotype of *Sabanejewiabulgarica* with karyotypes of other species of this genus analysed so far (Table 1). It is evident that the karyotype of *S.bulgarica* shows some apparent similarities with those of congeneric species: i) the same diploid chromosome number 2n = 50, ii) the low number of m chromosomes (two pairs), iii) the numbers of sm chromosomes (six pairs) and st to a chromosomes (17 pairs), iv) likely C-banding pattern correspond to those of *S.balcanica* (Ráb et al. 1991). The comparison also demonstrates some differences in karyotype structure, which, however, need explanation. Lodi and Marchioni (1980) and Vasil’ev (1985) distinguished the categories of st and a chromosome while Vasil’eva and Vasil’ev (1988), Ráb et al. (1991) and Vasil’ev and Vasil’eva (1994, 2019) did not recognize these morphological types of chromosomes as different ones but combined them together. This is especially due to the presence of small acrocentric-like chromosomes with their centromere position ranging gradually from subterminal to nearly terminal which makes difficult the precise assignment of these chromosomes to particular morphological types. The possible interspecific differences in proportion of such st and a chromosomes are in addition masked by the effect of chromosome arms contraction during mitosis due to the effect of timing and dose of colchicine treatment. Thus, it is difficult to interpret the karyotype descriptions of different authors.

In conclusion, the karyotype of *S.bulgarica* from the Danube Delta is very similar to karyotypes of other *Sabanejewia* species in respect of the chromosome number and morphological types of chromosomes, and is nearly identical with karyotype of *S.balcanica* from the Northern Carpathian range in Slovakia. Further detailed cytotaxonomic as well as other genetic surveys of populations of *Sabanejewia* throughout its distribution will answer problems of systematics of Danubian golden loaches.

**Table 1.** Diploid chromosome numbers (2n) and karyotype structure of karyologically studied species of genus *Sabanejewia*. Types of chromosomes: m –metacentric, sm – submetacentric, st – subtelocentric, a – acrocentric.

Species	2n	Karyotype characteristics				References
		m	sm	st	a	
*S.larvata*	50	2	3	11	9	Lodi and Marchioni 1980
*S.caspia*	50	2	3	11	9	Vasil’ev 1985; Vasil’ev and Vasil’eva 1994
*S.kubanica*	50	3	7	15		Vasil’eva and Vasil’ev 1988
*S.aurataaurata*	50	3	6	16		Vasil’ev and Vasil’eva 1994
*S. (aurata) balcanica*	50	2	6	17		Ráb et al. 1991
*S. (aurata) balcanica*	50	2	6	17		Vasil’ev and Vasil’eva 1994
*S.baltica*	50	2	8	15		Boroń 2000
*S.bulgarica*	50	2	6	17		This study
